# Behavioral Changes in Patients With Diabetes During the COVID-19 Pandemic in Saudi Arabia: A Cross-Sectional Study

**DOI:** 10.7759/cureus.34956

**Published:** 2023-02-14

**Authors:** Bader Fatani, Nawaf K Alfhaed, Aseel A Alkhemshi, Aseel A Alomireeni, Maha S Albarrak, Mohammed F Alquhayz, Saeed G Alzahrani

**Affiliations:** 1 Dentistry, College of Dentistry, King Saud University, Riyadh, SAU; 2 Medicine and Surgery, Imam Mohammad Ibn Saud Islamic University, Riyadh, SAU; 3 Medicine and Surgery, College of Medicine, King Saud University, Riyadh, SAU; 4 Public Health, Imam Mohammad Ibn Saud Islamic University, Riyadh, SAU

**Keywords:** coronavirus, diabetes, diabetes type 1, diabetes type 2, covid-19

## Abstract

On March 2, 2020, the first case of coronavirus disease 2019 (COVID-19) was dedicated in Saudi Arabia. The government established partial quarantine, and all precautions were mandatory on March 23, 2020. This in turn forced people to spend more time at home, leading to changes in the physical activity and dietary habits of individuals. In this study, we aimed to assess the behavioral changes of Saudi diabetic individuals during the COVID-19 pandemic and measure the effectiveness of the behavioral changes of Saudi diabetic individuals during the COVID-19 pandemic on the control of the glucose level.

A cross-sectional study was conducted through an online questionnaire sent to diabetic patients in Riyadh, Saudi Arabia. Participants ranging from 18 years old and above of both genders were selected. Diabetic patients (type 1 and 2) were included. All collected data for this study were analyzed using Stata 17 (StataCorp LLC, College Station, Texas, USA). A total of 223 people responded. For 45% of individuals, doctor visits significantly decreased (p<0.05), and the percentage of doctor visits also decreased for individuals who previously visited their doctor more regularly. Furthermore, the number of hours spent on tablets increased significantly during COVID-19 (p<0.05). Our findings demonstrate that there are no significant changes in lifestyle factors or glycemic control during the pandemic compared with the pre-pandemic year in individuals with diabetes. However, the rate of patient visits to the doctor was lower than pre-pandemic, with an increase in the rate of physical activity.

## Introduction

Coronaviruses (CoVs) are among the most common causes of human respiratory infections; it includes the severe acute respiratory syndrome (SARS) coronavirus (SARS-CoV) and Middle East respiratory syndrome coronavirus (MERS-CoV), which are two of the most highly transmissible and pathogenic viruses that emerged in humans [1]. Coronavirus disease 2019 (COVID-19) is an infectious disease caused by the severe acute respiratory syndrome coronavirus 2 (SARS-CoV-2), which was discovered at the end of 2019 in China and later became one of the major outbreaks throughout history [2]. This virus has been a current issue due to rapid transmission from person to person through close contact. Therefore, precautionary measures such as wearing face masks, social restrictions, and hand hygiene are essential to overcome and control the outbreak [3].

The first case of COVID-19 infection recorded in Saudi Arabia was on March 2, 2020. Following this case, on March 8, the Saudi government suspended education to avoid more substantial outbreaks, and on March 23, the Saudi government established partial quarantine, and all precautions were mandatory. These precautions constrained people to stay at home, leading to changes in physical activities and dietary habits of individuals. Of particular concern is that the analyses of fatalities and patients who have been infected with a severe course of COVID-19 or have not been infected have shown that people with chronic diseases are more vulnerable to changes in behaviors and habits [2]. Further medical research has revealed that people with diabetes are at a higher risk of developing severe COVID-19 [2]. Moreover, extensive research has shown that there is a significant rise in diabetes prevalence in Saudi Arabia in the last two decades, which has been accompanied by changes in lifestyle, and its rate has become one of the highest worldwide [4].

Since there are approximately 463 million adults with diabetes in the last statistic in 2019 in the Kingdom of Saudi Arabia (KSA) and it is well known that individuals with diabetes are at a higher risk because of their underlying disease and the prevalence of comorbidity, evaluating the effects of the COVID-19 pandemic on these diabetic patients plays a critical role in the maintenance of public health and evaluating the behavioral changes of these patients as a result of the pandemic. Regarding lifestyle changes, social constraints can lead to a significant decrease in levels of moderate to vigorous physical activity as well as an increase in time spent on sedentary behaviors such as watching television or time consumed on social media, which considerably may influence glycemic control and weight gain [5].

In addition, there is a lack of published data in Saudi Arabia that has paid attention to the role of the psychological impact of the COVID-19 pandemic on diabetic patients. For this reason, this research aims to understand and assess the effect of the COVID-19 pandemic on lifestyle, behavioral changes, and glucose level control among Saudi diabetic individuals. Nevertheless, such actions can either support diabetic patients’ health or increase their vulnerability to diseases and viruses. Patients and care providers should focus together on how to optimize lifestyle from the time of the initial comprehensive medical evaluation to examine changes in individual behavior, body weight, and glycosylated hemoglobin (HbA1c) levels of diabetic patients over the period of the proclamation of a state of emergency to see how this declaration affects their lifestyle and glycemic control. Previous studies have discussed the effect of the COVID-19 pandemic on the population’s daily lifestyle. However, there are no current data explaining the behavioral changes of Saudi diabetic individuals during the COVID-19 pandemic.

## Materials and methods

This research design is cross-sectional to assess the behavioral changes in patients with diabetes during the COVID-19 pandemic in Riyadh. Informed consent was clear and indicated the purpose of the study and the right of the participant to withdraw at any time without any obligation toward the study team. Participants were given no incentives or rewards, and the participant’s right to confidentiality was respected. Participants were included if they were Saudi, diabetic (type 1 and 2), and above 18 years old. On the other hand, non-Saudi and non-diabetic patients and those less than 18 years were excluded.

A structured questionnaire was used as a study tool. This tool was developed after accessing relevant studies conducted in Saudi Arabia and elsewhere. The final version of the questionnaire is classified into five main sections. The first section contained socioeconomic background characteristics questions, the second section included information about diabetes mellitus (DM) such as the duration of the disease and the type of DM, the third section was about DM and its pattern, and the fourth and fifth sections included questions about knowledge and practice.

The study was carried out in Riyadh, Saudi Arabia, from August 2021 to January 2022.

Statistical analysis

The primary behavioral factors that our study aims to examine are smoking, doctor visits, physical activities, eating habits, sleeping hours, sugar levels, and tablet use. We reported the descriptive values (counts and proportions) for each factor and calculated the relative proportional change. We examined the significance of the change in participants’ responses to the selected factor before and during COVID-19 using the chi-square test. We selected a p-value of <0.05 to determine the significance cutoff value. All data management and statistical analysis were performed using Stata 17 (StataCorp LLC, College Station, Texas, USA).

## Results

During the data collection, 223 people responded, with a mean age of 33.6 years. The majority (72.6%) were female, and 83.4% had previously been diagnosed with diabetes. More than half of those surveyed do not know what type of diabetes they have. The most commonly known type of diabetes reported by our study participants was type 1. Around 56% stated that they had a family history of diabetes. In terms of socioeconomic factors, 40.5% of the participants were unemployed, 59.1% had completed their undergraduate education, and only 5.5% had a postgraduate degree. Although 84.2% of the total sample was vaccinated, nearly half of the participants reported an increase in anxiety during the COVID-19 pandemic. At the time of filling out questionnaires, the average body weight of the participants was 65 kg (SD: 15.8 kg), with 40% reporting that their weight did not change during the COVID-19 pandemic and 60% responding evenly, saying it either increased or decreased (Table 1).

**Table 1:** Characteristics of the study population SD: standard deviation, DM: diabetes mellitus, COVID-19: coronavirus disease 2019

Characteristic		Number (%)
Sex	Female	162 (72.6)
	Male	61 (27.4)
Age in years, mean (SD)	33.6 (14.7)	
Diabetic patient	Yes	186 (83.4)
	No	37 (16.6)
DM type	Type 1	62 (24.4)
	Type 2	42 (16.5)
	Unknown	150 (59.1)
Family history of DM	Yes	143 (56.3)
	No	69 (27.2)
	Not sure	42 (16.5)
Marital status	Single	146 (57.5)
	Married	85 (33.5)
	Other	23 (9)
Education	Postgraduate	14 (5.5)
	Undergraduate	150 (59.1)
	High school	64 (25.2)
	Other	26 (10.2)
Job status	Employed	128 (50.4)
	Unemployed	103 (40.5)
	Retired	23 (9.1)
Insured (yes)	118 (46.5)	
Had COVID-19 before (yes)	91 (35.8)	
Vaccinated for COVID-19 (Yes)	210 (84.2)	
Anxiety increased during COVID-19 (yes)	133 (52.4)	
Weight in kg, mean (SD)	65 (15.8)	
Weight gained during COVID-19	>3 kg	33 (13)
	1-3 kg	45 (17.7)
	Did not change	102 (40.2)
	<3 kg	33 (13)
	<1-3 kg	41 (16.1)

Table 2 displays participant responses to a set of variables that might indicate behavioral changes prior to and during the COVID-19 pandemic. Smoking habits, physical activities, eating habits, and sugar levels did not significantly change.

**Table 2:** Behavioral changes before and during the COVID-19 pandemic COVID-19: coronavirus disease 2019

	Before	During	Proportional change (%)	p-value
Smoking				0.7223
Less than once a week	12 (5.4)	16 (7.2)	+1.8	
Never	182 (81.6)	184 (82.5)	+0.9	
At least once a week	11 (4.9)	8 (3.6)	-1.3	
Daily	18 (8.1)	15 (6.7)	-1.4	
Doctor visit (annually)				0.0001
>5 times	45 (20.8)	25 (11.2)	-9.6	
Never	35 (15.7)	102 (45.7)	+30.0	
Once only	60 (26.9)	47 (21.1)	-5.8	
2-5 times	83 (37.2)	49 (22)	-15.2	
Physical activity (per week)				0.1427
1 time	37 (16.6)	27 (12.1)	-4.5	
2 times	45 (20.2)	36 (16.2)	-4.0	
3 times	34 (15.2)	30 (13.4)	-1.8	
4 times	17 (7.8)	19 (8.5)	+0.7	
5 times	13 (5.8)	11 (4.9)	-0.9	
6 times	9 (4)	14 (6.3)	+2.3	
Every day	13 (5.8)	30 (13.4)	+7.6	
Never	55 (24.6)	56 (25.2)	+0.6	
Eating habits				
Eating fruits (per week)				0.5609
2-4 times	56 (25.1)	55 (24.6)	-0.5	
5-6 times	15(6.7)	23 (10.3)	+3.6	
More than 6 times	10 (4.5)	12 (5.4)	+0.9	
Daily	20 (9)	19 (8.5)	-0.5	
Once	52 (23.3)	42 (18.8)	-4.5	
Less than once	50 (22.4)	49 (22)	-0.4	
Never	20 (9)	23 (10.4)	+1.4	
Eating vegetables (per week)				0.6711
2-4 times	67 (30)	63 (28.3)	-1.7	
5-6 times	22 (9.9)	33 (14.8)	+4.9	
More than 6 times	13 (5.8)	13 (5.8)	+0.0	
Daily	19 (8.5)	21 (9.4)	+0.9	
Once	43 (19.3)	44 (19.7)	+0.4	
Less than once	43 (19.3)	33 (14.8)	-4.5	
Never	16 (7.2)	16 (7.2)	+0.0	
Eating sweets (per week)				0.5842
2-4 times	58 (26)	52 (23.3)	-2.7	
5-6 times	21(9.5)	16 (7.2)	-2.3	
More than 6 times	17 (7.6)	15 (6.7)	-0.9	
Daily	15 (6.7)	11 (4.9)	-1.8	
Once	42 (18.8)	56 (25.1)	+6.3	
Less than once	52 (23.3)	49 (22)	-1.3	
Never	18 (8.1)	24 (10.8)	2.7	
Sleeping hours (per day)				0.0001161
<7	84 (37.7)	71 (31.8)	-5.9	
7-9	112 (50.2)	89 (39.9)	-10.3	
>9	27 (12.1)	63 (28.3)	+16.2	
Sugar level self-screening				0.2571
Good	48 (21.5)	34 (15.2)	-6.3	
Very good	50 (22.4)	41 (18.4)	-4.0	
Never	65 (29.1)	79 (35.5)	+6.4	
Acceptable	21 (9.4)	23 (10.3)	0.9	
Excellent	39 (17.6)	46 (20.6)	+3.0	
Tablet use (hours per day)				0.0002108
<1	39 (17.5)	37 (16.6)	-0.9	
>8	50 (22.4)	74 (33.2)	+10.8	
2-4	57 (25.6)	25 (11.2)	-14.4	
4-6	51 (22.9)	44 (19.7)	-3.2	
6-8	26 (11.6)	43 (19.3)	+7.7	

Some habits, including physical activity, had only slight changes during the COVID-19 pandemic, with the percentage of people who exercise every day rising by 7.6%. On the other hand, for 45% of individuals, doctor visits significantly decreased to almost none over the study period (p<0.05). The percentage of doctor visits in person also decreased for individuals who previously visited their doctor more regularly, by -9.6% for >5 visits and -15% for those who previously made 2-5 visits annually. Similarly, participants reported that during the COVID-19 pandemic, the proportion of times they slept for more than nine hours increased by about 16% compared to the pre-COVID-19 period. Furthermore, the number of hours spent on tablets increased significantly during the COVID-19 pandemic (p<0.05). The differences between tablet use, sleeping hours, and doctor visits among the sample are shown in Table 3.

**Table 3:** Differences between tablet use, sleeping hours, and doctor visits among the sample

	Crude (unadjusted)	p-value	Adjusted for age	p-value	Adjusted for age and sex	p-value	Adjusted for age, sex, and education	p-value
Tablet use	1.77 (1.47-2.12)	<0.001	1.74 (1.44-2.09)	<0.001	1.74 (1.45-2.09)	<0.001	1.72 (1.43-2.07)	<0.001
Sleeping hours	2.01 (1.46-2.76)	<0.001	1.96 (1.42-2.71)	<0.001	1.95 (1.41-2.69)	<0.001	1.95 (1.41-2.69)	<0.001
Doctor visits	1.16 (0.89-1.52)	0.272	1.16 (0.89-1.52)	0.272	1.17 (0.89-1.54)	0.265	1.17 (0.89-1.55)	0.250

## Discussion

The COVID-19 lockdown was found to have a negative impact on the adherence of diabetes patients to their medication regimens in this study. The lockdown also resulted in significant changes in behavioral habits such as smoking and visiting the doctor, which matched the findings of a study that found that a high percentage of participants with diabetes suspected the risk of COVID-19 infection and increased their smoking and drinking during the pandemic [6]. Most of the patients’ physical activity and sleep patterns were affected by the lockdown, while most patients’ eating habits were unaffected by the lockdown.

This is the first Saudi Arabian study to look at the effect of the COVID-19 lockdown on Riyadh’s diabetic population. Both people and the healthcare system are heavily burdened by diabetes mellitus. The risk of complications and death can be greatly increased if blood glucose levels are not adequately maintained. As a result, patients’ compliance with their medical treatment and a healthy lifestyle is vital.

The COVID-19 lockdown negatively affected the adherence of diabetes patients to their medication schedules, which matched with the study that aimed to see how the coronavirus disease quarantine affected diabetes patients in Jeddah, Saudi Arabia [7], in regard to medication adherence, lifestyle behaviors, and the overall quality of life. The study showed that patients’ levels of medication adherence and positive lifestyle practices were considerably reduced during the lockdown. These findings underscore the need for encouraging diabetes patients to maintain good lifestyle behaviors and use telemedicine during lockdowns to achieve optimal blood glucose control and avoid the risk of consequences.

The study aimed to look at how diabetic patients changed their behaviors during the COVID-19 pandemic and see how this emergency incident affected diabetic patients’ lifestyles and glucose management [8]. The findings of the study showed that reduced levels of physical activity throughout the state of emergency had a negative impact on glucose control. Preserving or enhancing eating habits, regardless of changes in the level of physical activity, may contribute to greater glycemic control in diabetes patients. This study showed that the lockdown had a negative effect on the physical activity and sleeping hours of most of the patients and a minimal effect on the eating habits of most of the patients, and this also contributed to greater glycemic control in diabetes patients. This matched the results of a study that showed that lockdown improves glycemic control in persons with type 1 diabetes [9]. While the lockdown was a source of concern for several people with type 1 diabetes, their research reveals that it is also a chance to make good behavioral changes.

This study showed that behavioral changes such as smoking and visiting doctors were found to be significantly increased due to the COVID-19 lockdown. The findings of a previous study by Malta et al. that aimed to describe lifestyle changes in terms of tobacco and alcohol usage, food consumption, and physical exercise during the time of social restriction caused by the COVID-19 disease outbreak showed that there is a deterioration in lifestyles and an increase in health risk behaviors [5]. Moreover, Hosomi et al. aimed to look into the acute impacts of COVID-19 on individuals with type 1 diabetes on their ways of living and metabolic markers and showed that the glycemic control of type 1 diabetes patients was much worse than the prior year [10]. As the epidemic continues, people with type 1 diabetes should pay greater attention to stress and lifestyle factor management. A study by Musche et al. also showed that diabetic patients have higher COVID-19-related dread, increased risk perception, and behavioral alterations [2]. Moreover, Takahara et al. showed that throughout the COVID-19 epidemic, a significant number of outpatients with diabetes underwent lifestyle adjustments [11].

Limitation

No validated assessment tool for COVID-19-related fear was available at the time the study was conducted. Using one single item, an online survey, the answers to the included questions depended predominantly on the patients’ honesty and subjective opinions; this may have affected outcome validity. It was also one of the limitations that we faced. We were surprised by the large number of patients suffering from diabetes who could not distinguish which type they had, either type 1 or type 2, noting that the second type is the most common in Saudi Arabia. The limitation also included response bias, a small population, and a lack of generalizability to the overall population given the survey was conducted in an urban setting. The population in rural areas might have differed in their behaviors during and before the pandemic. Moreover, behaviors varied among people with and without employment; thus, this should be assessed in future studies.

Recommendation

We advise that people with diabetes should give greater attention to stress and lifestyle factor management. Preserving or enhancing eating habits, regardless of changes in the level of physical activity, may contribute to greater glycemic control in diabetes patients. There was an increase in physical activity. However, more studies need to be carried out to investigate other factors that increase weight gain among the population, such as stress, anxiety, sleeping hours, and eating habits. We recommend investigating the effect of televisits on patients with diabetes and assessing all reasons for anxiety in these patients in the future.

## Conclusions

In conclusion, the COVID-19 pandemic has hugely influenced the population, particularly individuals who are diabetic, and their everyday life. Our findings demonstrate that a percentage of the participants reported their anxiety during the COVID-19 pandemic. Moreover, there are no significant changes in lifestyle factors or glycemic control during the pandemic compared with the pre-pandemic year in individuals with diabetes. However, the rate of patient visits to the doctor was lower than pre-pandemic, with an increase in the rate of physical activity. Therefore, we suggest more studies to follow up with diabetics in crises such as the COVID-19 pandemic to determine the best methods to reduce the negative effects and benefit from them by increasing the rate of exercise performance, controlling the level of glucose by strengthening telemedicine networks, and reducing the complications of diabetes.
